# Supplementary material for: Distribution of OGTT-Related Variables in Patients with Cystic Fibrosis from Puberty to Adulthood: An Italian Multicenter Study
Source: J Pers Med. 2023 Mar 3;13(3):469. doi: 10.3390/jpm13030469 (PMC10056682; doi:10.3390/jpm13030469)
Supplement: Supplementary file 1 [file jpm-13-00469-s001.zip › jpm-2185690-supplementary.pdf]

Distribution of OGTT-related variables in patients with Cystic Fibrosis

Supplementary Materials

Comparison with general population

Study	Country	Sample size	Age range (years)	Female patients
Ford (2006)	USA	1791	12-19	860 (48%)
Koesterweber (2014)	Europe	927	12-17	506 (55%)
Peplies (2014)	Europe	7074	3-11	3434 (49%)
Tohidi (2014)	Iran	309	24-83	185 (60%)

Table S1: Sample characteristics of studies providing reference values for fasting insulin

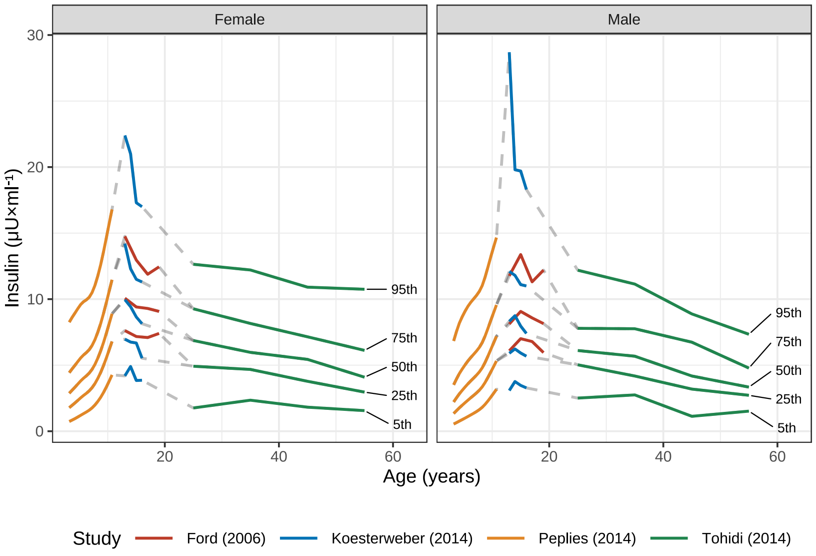

Figure S1: Centiles of fasting insulin available in the literature, with dashed interpolated lines between studies

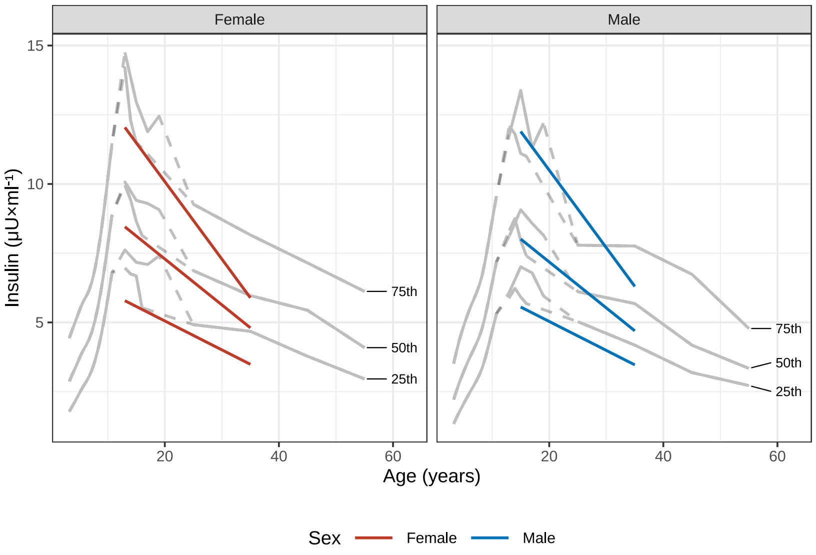

Figure S2: Comparison between cystic fibrosis and general population quartiles of fasting insulin.

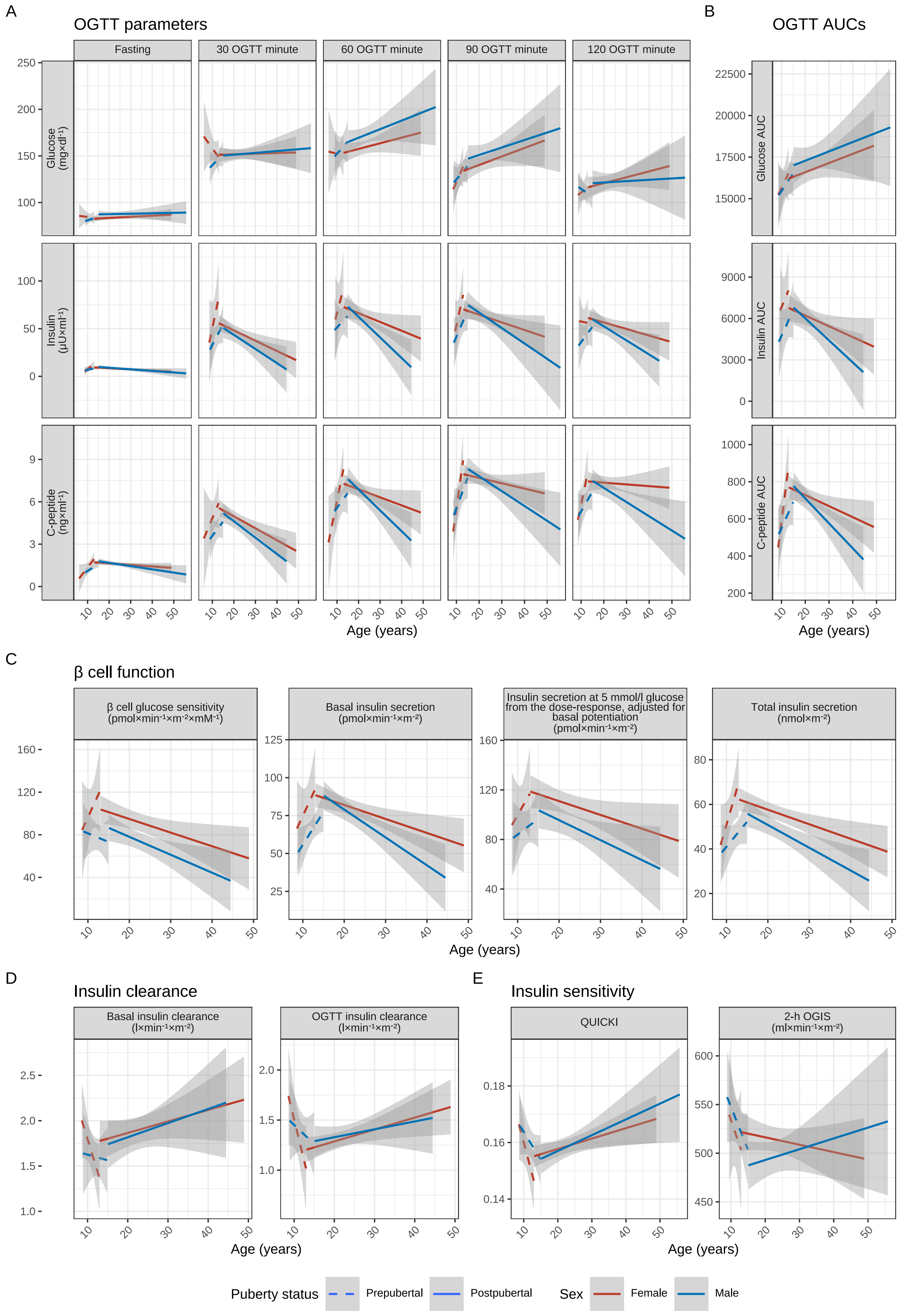

Figure S3: Within sex linear trends before and after puberty of OGTT parameters, beta cell function, insulin clearance, and insulin sensitivity.

Post-puberal reference values

Multiple imputation by chained equations

We used MICE to create 100 complete versions of data by replacing missing values with plausible data values (Van Buuren 2018; White, Royston, and Wood 2011). The imputed variables were (in order of decreasing missingness):

Variable	Missing count
HbA1c	130
FVC	95
Insulin AUC	94
FEV1	89
OGIS 120	62
Basal insulin clearance	50
CRP	50
Insulin 30	44
HOMA-IR	40
Fasting insulin	40
QUICKI	40
Insulin 90 minutes	36
Insulin 60 minutes	35
Insulin 120 minutes	34
Total insulin clearance	24
C-peptide AUC	21
Glucose sensitivity	19
Insulin secretion at 5 mmol/l glucose from the dose-response, adjusted for basal potentiation	18
C-peptide 30	15
C-peptide 90	15
C-peptide 120	15
C-peptide 60	14
C-peptide 0	13
Glucose AUC	5
Glucose 90	4
Glucose 60	2
Glucose 120	2
Glucose 30	1

The complete variables were:

- sex (discrete)
- center (discrete)
- age (continuous)
- weight (continuous)
- height (continuous)
- fasting glucose (continuous)
- pancreatic insufficiency (binary)

Because most variables had non-Gaussian distributions, imputation was performed using predictive mean matching with 5 knots on 100 multiple imputation datasets (Van Buuren 2018; White, Royston, and Wood 2011). Trace plots of imputed values against iteration numbers were used to assess the stationarity of the chains (Van Buuren 2018; White, Royston, and Wood 2011). The imputer and the analyst were the same person, and the scope of the MI model was narrow, i.e., it was devised for testing only the present study hypothesis (Sterne et al. 2009). The linearity of association of the outcome with age was tested using multivariable fractional polynomials (powers -2, -1, -0.5, 0 (=logarithm), 0.5, 1, 2 and 3) for multiple imputation (Morris et al. 2015). All relationships were linear. We calculated and plotted the marginal probabilities of each variable corresponding to the 25th, 50th and 75th percentile (Klein 2015; Williams 2012). MICE was performed using Stata 17.0 (Stata Corporation, College Station, TX, USA).

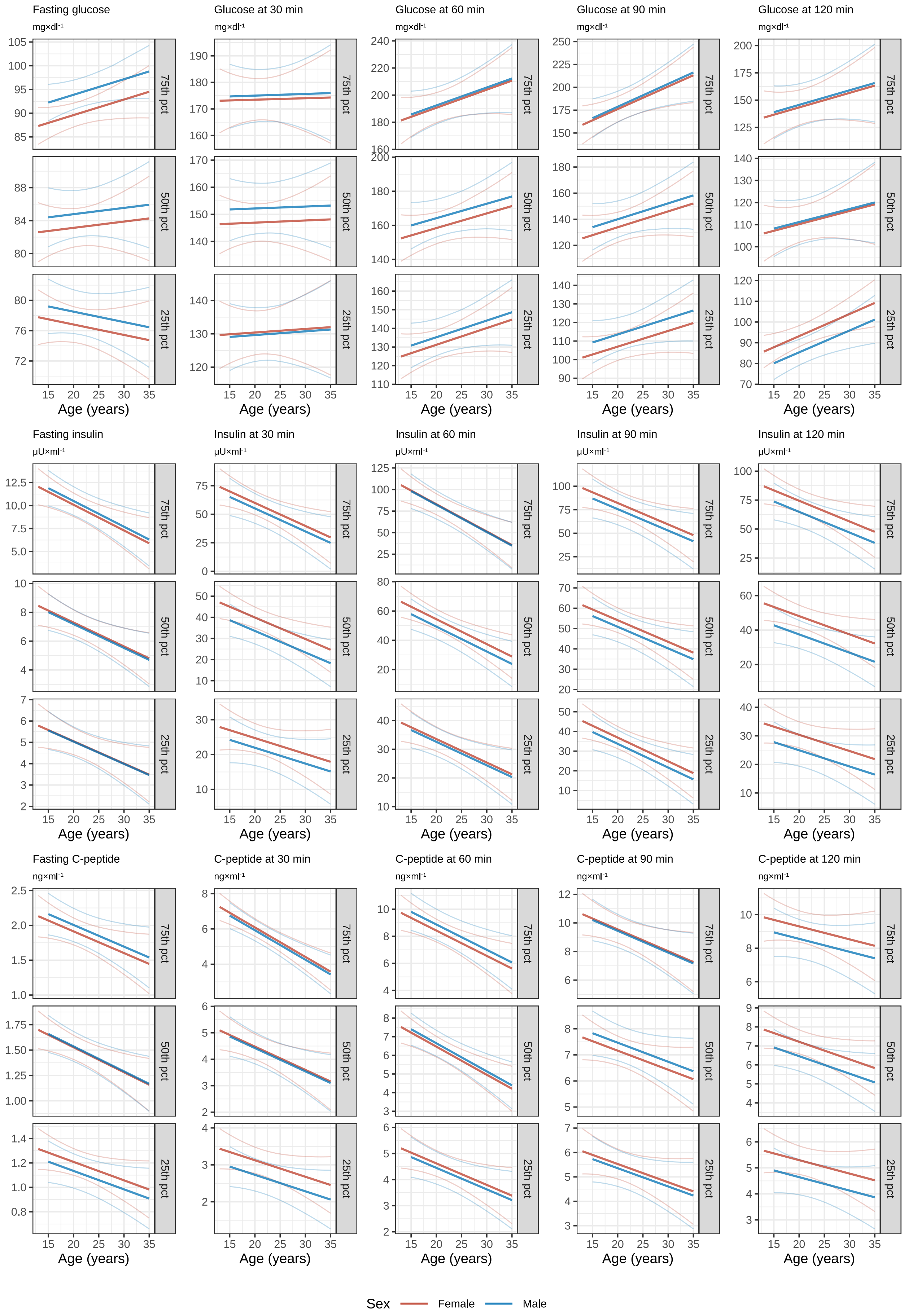

Figure S4: Point estimates and 95% confidence intervals from quantile regression of OGTT parameters.

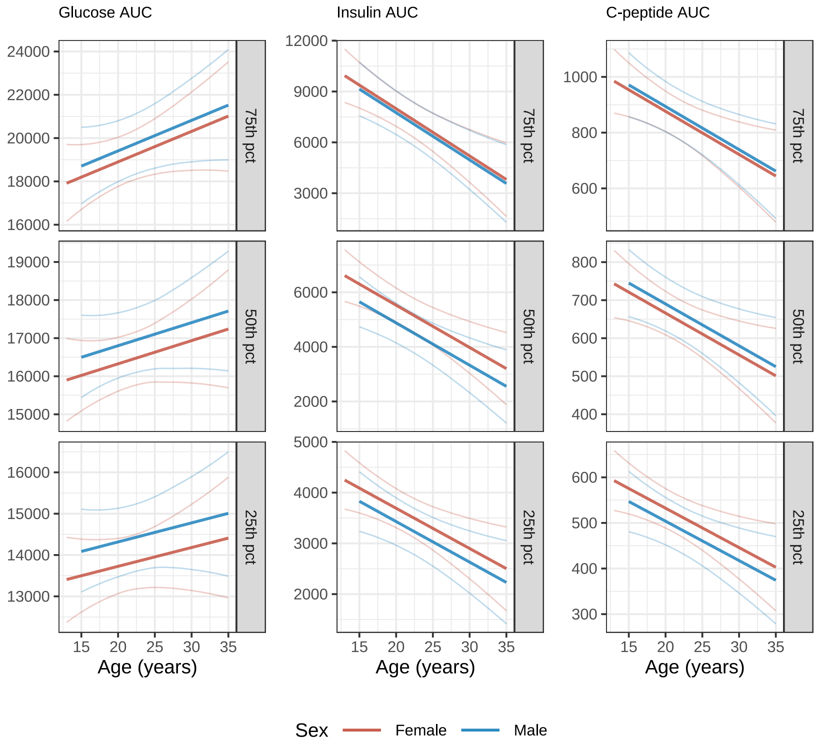

Figure S5: Point estimates and 95% confidence intervals from quantile regression of OGTT AUCs.

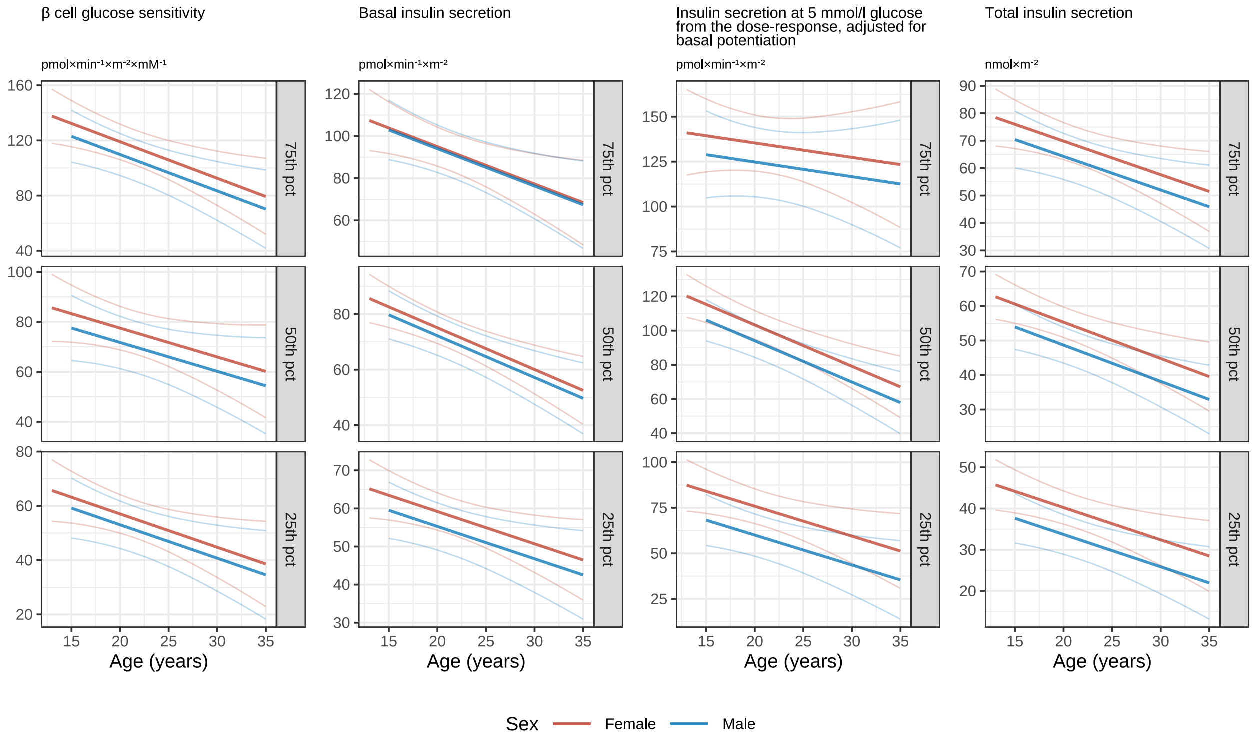

Figure S6: Point estimates and 95% confidence intervals from quantile regression of beta cell function parameters.

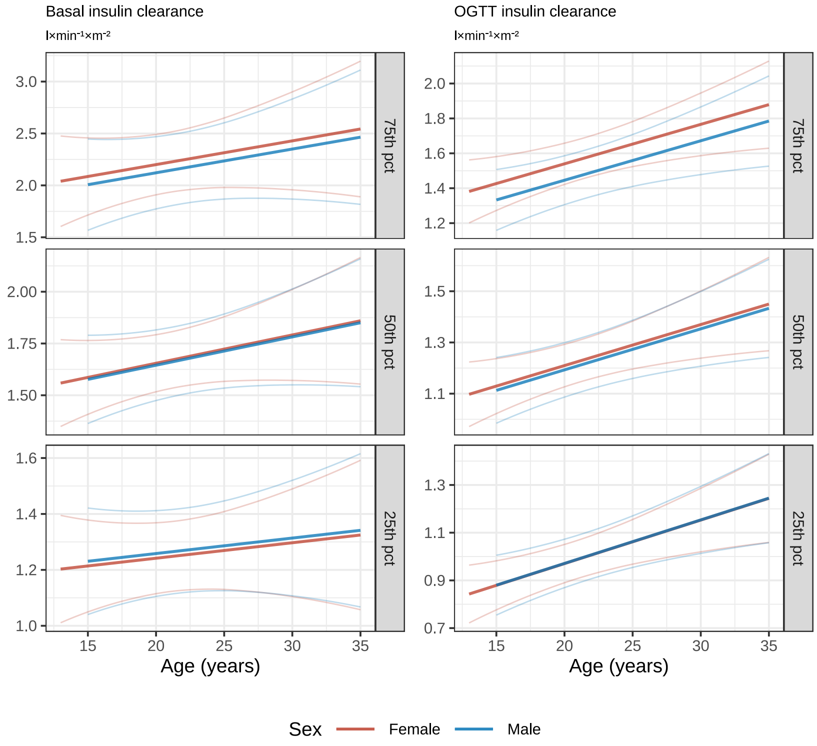

Figure S7: Point estimates and 95% confidence intervals from quantile regression of insulin clearance parameters.

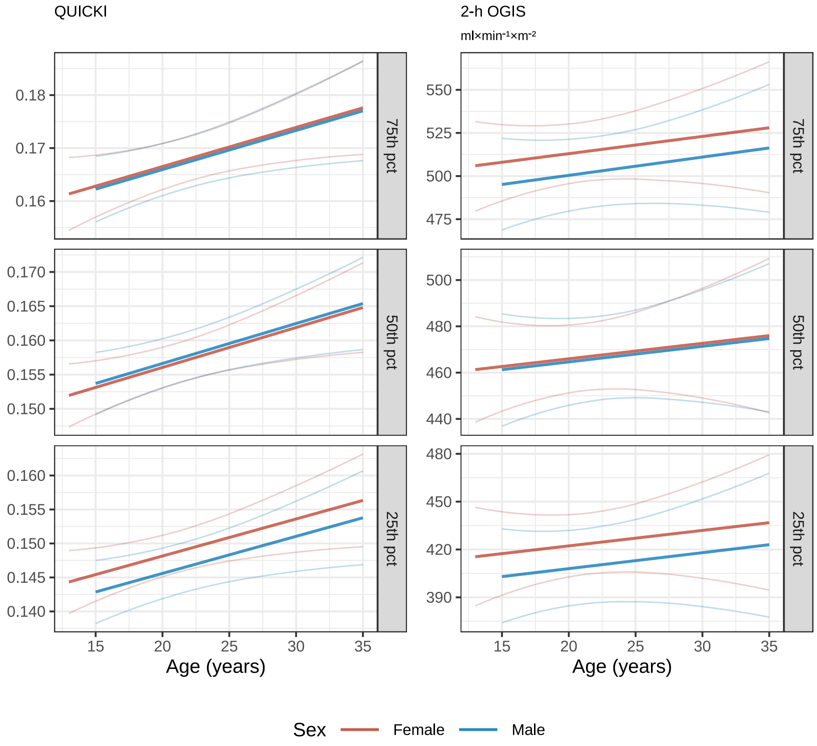

Figure S8: Point estimates and 95% confidence intervals from quantile regression of insulin sensitivity indices.

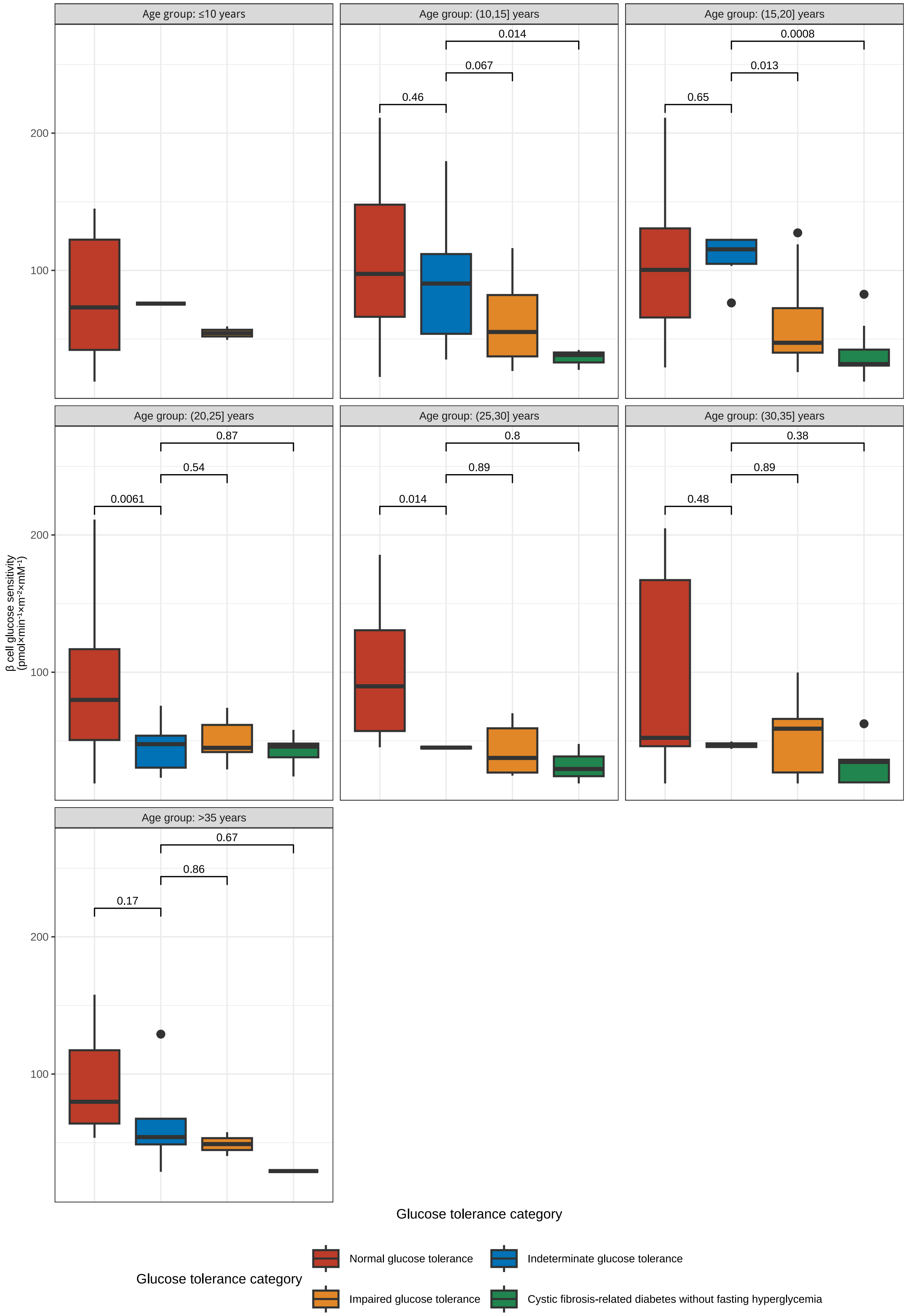

Figure S9: [illegible] cell glucose sensitivity comparison (Wilcoxon test) across glucose tolerance category, stratified by age groups.

References

Ford, Earl S., Chaoyang Li, Giuseppina Imperatore, and Stephen Cook. 2006. "Age, Sex, and Ethnic Variations in Serum Insulin Concentrations Among u.s. Youth." Diabetes Care 29 (12): 2605–11. https://doi.org/10.2337/dc06-1083.

Klein, Daniel. 2015. "MIMRGNS: Stata Module to Run Margins After Mi Estimate (Statistical Software Components)." Chestnut Hill: Boston College Department of Economics. Zugriff Am 18: 2015.

Koester-Weber, Thabata, Jara Valtueña, Christina Breidenassel, Laurent Beghin, Maria Plada, Sara Moreno, Inge Huybrechts, et al. 2014. "Reference Values for Leptin, Cortisol, Insulin and Glucose, Among European Adolescents and Their Association with Adiposity: The HELENA Study." Nutr. Hosp. 30 (5): 1181–90.

Morris, Tim P, Ian R White, James R Carpenter, Simon J Stanworth, and Patrick Royston. 2015. "Combining Fractional Polynomial Model Building with Multiple Imputation." Statistics in Medicine 34 (25): 3298–3317.

Peplies, J, and D Jiménez-Pavón, S C Savva, C Buck, K Günther, A Fraterman, P Russo, et al. 2014. "Percentiles of Fasting Serum Insulin, Glucose, HbA1c and HOMA-IR in Pre-Pubertal Normal Weight European Children from the IDEFICS Cohort." Int J Obes 38 (S2): S39–47. https://doi.org/10.1038/ijo.2014.134.

Sterne, Jonathan A C, Ian R White, John B Carlin, Michael Spratt, Patrick Royston, Michael G Kenward, Angela M Wood, and James R Carpenter. 2009. "Multiple Imputation for Missing Data in Epidemiological and Clinical Research: Potential and Pitfalls." BMJ 338. https://doi.org/10.1136/bmj.b2393.

Tohidi, Maryam, Asghar Ghasemi, Farzad Hadaegh, Arash Derakhshan, Abdolreza Chary, and Fereidoun Azizi. 2014. "Age- and Sex-Specific Reference Values for Fasting Serum Insulin Levels and Insulin Resistance/Sensitivity Indices in Healthy Iranian Adults: Tehran Lipid and Glucose Study." Clinical Biochemistry 47 (6): 432–38. https://doi.org/10.1016/j.clinbiochem.2014.02.007.

Van Buuren, Stef. 2018. Flexible Imputation of Missing Data. CRC press.

White, Ian R., Patrick Royston, and Angela M. Wood. 2011. "Multiple Imputation Using Chained Equations: Issues and Guidance for Practice." Statistics in Medicine 30 (4): 377–99. https://doi.org/https://doi.org/10.1002/sim.4067.

Williams, Richard. 2012. "Using the Margins Command to Estimate and Interpret Adjusted Predictions and Marginal Effects." The Stata Journal 12 (2): 308–31.
